# Long-short term memory networks for modeling track geometry in laser metal deposition

**DOI:** 10.3389/frai.2023.1156630

**Published:** 2023-06-21

**Authors:** Martina Perani, Ralf Jandl, Stefano Baraldo, Anna Valente, Beatrice Paoli

**Affiliations:** ^1^Laboratory for Web Science, Department for Research and Services, Fernfachhochschule Schweiz (FFHS), Brig, Switzerland; ^2^Automation Robotics and Machines Laboratory, Department of Innovative Technologies, University of Applied Science and Arts of Southern Switzerland (SUPSI), Lugano, Switzerland

**Keywords:** laser metal deposition, artificial intelligence, long-short-term-memory network, track height prediction, over-deposition, generalizable model, Inconel 718, process optimization

## Abstract

Modeling metal additive manufacturing processes is of great importance because it allows for the production of objects that are closer to the desired geometry and mechanical properties. Over-deposition often takes place during laser metal deposition, especially when the deposition head changes its direction and results in more material being melted onto the substrate. Modeling over-deposition is one of the necessary steps toward online process control, as a good model can be used in a closed-loop system to adjust the deposition parameters in real-time to reduce this phenomenon. In this study, we present a long-short memory neural network to model over-deposition. The model has been trained on simple geometries such as straight tracks, spiral and V-tracks made of Inconel 718. The model shows good generalization capabilities and can predict the height of more complex and previously unseen random tracks with limited performance loss. After the addition to the training dataset of a small amount of data coming from the random tracks, the performance of the model for such additional shapes improves significantly, making this approach feasible for more general applications as well.

## 1. Introduction

Additive manufacturing has gained traction in the last years thanks to its capability of manufacturing objects rapidly without the need of molds and with reduced material waste. Moreover, complex objects can be produced in one piece, often improving both mechanical properties and weight of the final parts (Godec et al., 2022). Laser Metal Deposition (LMD) is an additive manufacturing technique which uses a laser to melt metal powder onto a substrate. As the deposition head containing the laser moves, it leaves a solid metal track behind. As the deposition proceeds, an object will be created layer by layer (Schmidt et al., 2017).

**Figure 1:**
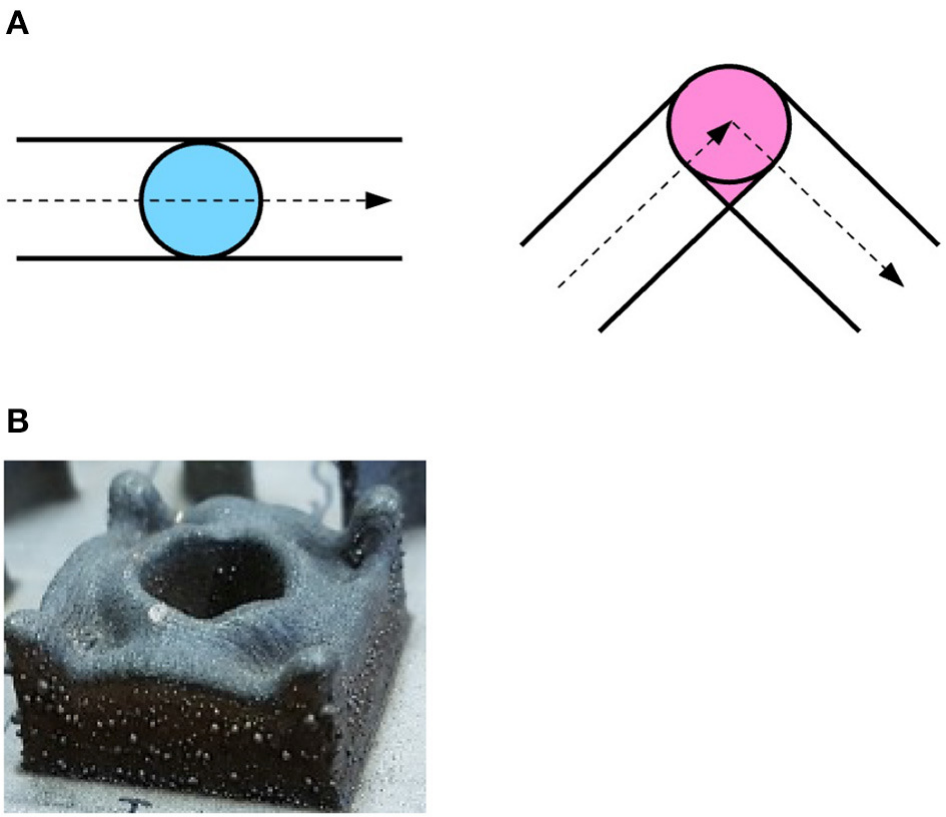
**(A)** Schematic view of the laser spot moving along a line (left) and changing direction at a corner (right). When making a corner, the laser spot covers the pink area in the right image more than once, resulting in more material deposited. **(B)** Over-deposition in a 3D object. The over-deposition in the corners of a single-layer adds up when manufacturing multi-layer parts, resulting in a significant deviation from the planned geometry.

The deposition process through LMD is very complex and the optimal deposition parameters when depositing new shapes or using new materials are unknown. In addition, the deposited parts often deviate from the planned geometry, which results in the need for intensive post-processing of the produced pieces in the best cases, and in total failure in the worst. One source of such deviations is the so-called *over-deposition*. This phenomenon occurs when the deposition head changes its direction. When the deposition head follows a straight line, the *melt pool* covers different points on the substrate with uniform duration within a layer (Figure 1A, left). When it changes direction, however, some parts of the substrate are covered for a longer time, due to the overlapping of track segments (Figure 1A, right), resulting in more material being deposited and therefore a larger height of the track at corners. Moreover, the deposition head decelerates before reaching a corner and then accelerates again after having changed its direction. This also contributes to an increase in the deposited material at corners. When a 3D object is printed, these effects sum up in subsequent layers, leading to significant deviations from the planned geometry (Figure 1B).

One way to address over-deposition is to modify the deposition parameters (such as laser power) when the deposition head changes its direction. During the deposition, the process is monitored, the layer height is predicted, and the parameters can be changed through a closed-loop feedback system (Johnson et al., 2020). A good model of the deposition process is therefore needed. Numerical models have been employed in the literature to predict the deposition outcomes, such as the geometrical properties of the produced parts. The drawback of this approach is the high computational load required to simulate the process. Even simplified models (i.e. models that do not consider fluid flow) provide a good prediction of the layer height, but require approximately 45 minutes for simulating one 60 mm long layer (Peyre et al., 2017), which makes this approach not feasible for dynamically adapting the deposition parameters during the deposition of real 3D parts. Artificial intelligence can provide a solution to this problem, as the computational load is shifted to the training of the model. The time needed to predict the process outcomes during deployment depends on the features used and the complexity of the models, but it is in the range of milliseconds, several orders of magnitudes smaller compared to numerical models (Dai et al., 2020).

Artificial intelligence has been employed in literature for predicting the height, porosity, and defects of cuboids of a Fe-Ni alloy deposited through LMD (Lee et al., 2023). In this work, the Shapley additive explanation approach has been applied to estimate the effect of different deposition parameters such as power, speed, and powder flux on the process outcomes. Understanding the relationships between these quantities is important to determine an optimal range of process parameters. However, LMD intrinsically produces sequential data, a feature which has not been considered in this study, as the model predicts the average height of the cuboid. Recurrent neural networks with gated recurrent units have been employed in Mozaffar et al. (2018) to predict the thermal history of the LMD process based on synthetic data generated by finite element simulations with added noise. This approach demonstrates that recurrent neural networks are a good choice to analyze sequential data in additive manufacturing. In our previous work (Perani et al., 2023) we predicted the height, width and cross-section of Inconel 718 single tracks (i.e. a single layer of material deposited along a straight line) using a deep-learning system based on convolutional neural networks. The advantage of this approach relies on the capability of the system of analyzing images of the melt pool acquired during the deposition. On the other side, even if the deposition process was followed in its temporal evolution (i.e. a height prediction for each acquired image frame have been produced), the time dependencies of the data could not be modeled with such a system. As only single tracks were employed, the generalization capabilities of the model had not been investigated. Height prediction of single- and multi-layer tracks based on features available offline has been performed in Knüttel et al. (2022a,b). Fully connected networks and XG Boost have been employed to make a prediction based on the process parameters and the geometrical properties of the laser trajectory. This work demonstrates that machine learning models are suitable to predict the geometrical features of more complex shapes, however, real-time monitored features like melt-pool images were not considered, making this model suitable for off-line process optimization but not for real-time process control.

In the present study, we propose a novel pipeline comprising data acquisition, feature engineering and a model architecture based on a Long-Short-Term-Memory (LSTM) network to predict the height of single-layer tracks deposited through LMD. A model suitable for industrial use should be able to generalize, i.e. to make predictions on objects and structures not seen during training. To address the generalization capability of the model, simpler structures like straight lines and V-shaped objects are used during the training, while more complex tracks presenting random corners have been used for testing the performance of the model. This paper is organized as follows: Section 2 describes the data and the pre-processing used for developing the model, as well as the additional features added to the acquired experimental data. The used network architecture is described as well. Section 3 focuses on the results obtained for predicting track height, and in Section 4 the main results are discussed in detail. Section 5 summarizes the most important results.

## 2. Materials and methods

For the present study, different shapes deposited through LMD with the nickel alloy Inconel 718 are considered. The diameter of the Inconel 718 powder is 77 ± 30 μm and the deposition takes place on a substrate of the same material at ambient initial temperature. The machine used for the deposition is a Lasedyne 430 with four deposition nozzles and Argon as carrier gas (4 l/min). An IDS UI-3070CP-C-HQ2 camera mounted on the deposition head has been used to record a top view of the melt pool area. During the deposition, different data are recorded with a frequency of 200 Hz:

- X, Y, and Z position of the deposition head;
- laser activation signal;
- images of the melt pool (400 x 400 pixels).

**Figure 2:**
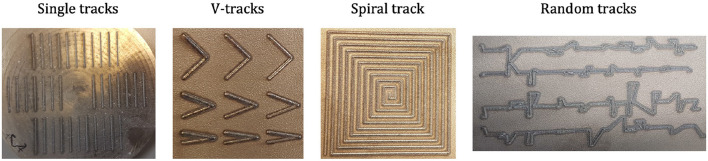
Different shapes used for training and testing the machine learning model: single tracks, spiral, V-tracks and random tracks.

After the deposition, the height, width, and cross-section of the deposited shapes have been measured using a GOM ATOS Core 200 fringe projection scanner. Different deposited shapes are considered in the present study: single tracks, V-shapes, spiral and single-layer random tracks. Figure 2 shows pictures of the deposited geometries. All deposited tracks are single-layer, and these shapes have been chosen as they are suitable to model over-deposition. The different tracks within the same shape type have been deposited using different parameters.

Table 1 summarizes the values used for the deposition speed, power, powder flux, and the resulting specific energies. All tracks have been deposited with a laser spot diameter of 1 mm. Single tracks are 20 mm long straight segments, and have been deposited at different nominal powers and speeds. Every possible combination of speed and power is present in the dataset, for a total of 36 tracks. V-shaped tracks have also been deposited for all the different combinations of power, speed, and angle, and each combination has been repeated three times, resulting in 27 different tracks. The side of the spiral track is 60 mm long with a step of 3 mm and the experiment has been repeated twice. The random tracks are about 90 mm long in x direction and span a maximum of 10 mm in y direction, and they are composed of randomly generated straight segments separated by random, sharp angles; they have been deposited with 300 W power and a cruise speed of 600 mm/min.

**Table 1:** Process parameters used for the different shapes.

**Track type**	**Deposition speed (mm/s)**	**Power (W)**	**Powder flux (g/s)**	**Angles**	**Specific energy (J/mm^2^)**
Single tracks	5–17.5, step 2.5	200-700, step 100	0.032	-	40–140
V-tracks	5.8, 10, 15	300	0.099	20°, 45°, 90°	20–52
Spiral	10	300	0.0825	90°	30
Random tracks	10	300	0.0825	random	30

**Figure 3:**
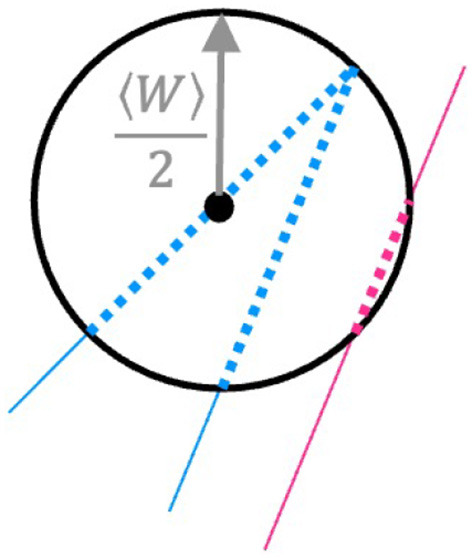
For each deposition point, a *W/2-neighbors* feature is created, which counts how many other deposition points (normalized to the data acquisition frequency) fall within the average track radius *W/2*. In the figure, the dotted line represents points falling within this neighborhood, and thus contributing to the W/2-neighbors feature (notice that also pink points from a nearby segment, although deposited at a distant time window, are included).

During a first preprocessing, the so-called *data fusion*, the data acquired during the deposition and the spatial measurements of the objects acquired thereafter (i.e. track height, width and cross-section) are synchronized and merged into one dataset suitable for machine learning purposes, as described in detail in (Perani et al., 2023). The resulting dataset for the single tracks (Baraldo, 2020), the spiral (Baraldo and Vandone, 2020a), the V-tracks (Baraldo and Vandone, 2020b) and random tracks (Baraldo and Giusti, 2021) are available under CC-BY 4.0 license. In order to model over-deposition, several new features have been added to the dataset. In addition to the deposition speed, its component *V*_*x*_ and *V*_*y*_ over the X and Y axes have also been considered. Two new features *W-neighbors* and *W/2-neighbors* have been created to account for the geometry of the tracks. Figure 3 describes the feature *W/2-neighbors*. For each data point at a specific time *t* (black dot in the figure), the deposition points within a radius of 〈*W*〉/2 are counted. 〈*W*〉 represents the average track width estimated from the single tracks. All deposition points in the past and in the future with respect to the time *t* that fall within this neighborhood are counted. Figure 3 shows an example: the deposition head is approaching a corner. All blue dots in the figure are within the set radius of 〈*W*〉/2 and are therefore counted. Sometime along the deposition, the head comes again close to this part of the track (pink solid line). Some points are again within the radius and are counted as well (pink dots). The W/2-neighbors feature is normalized with the acquisition frequency of the track, i.e.:


(1) $$\begin{array}{l} \text{W/2-neighbors}=\frac{\text{sum of the deposition points within r =}\langle W\rangle /2}{\langle \Delta t\rangle }. \end{array}$$

This feature can be evaluated before the deposition starts, as the trajectory of the deposition is determined beforehand. For this reason, this feature could also be used for more complicated geometries in a real-time setting. The feature *W-neighbors* is evaluated similarly, but considers points of the trajectory that fall within a 〈*W*〉 radius.

The dataset used for the training and testing of the machine learning model comprises:

- the deposition speed *V* computed from the X, Y, and Z position of the deposition head;
- the components of the speed vector *V*_*x*_ and *V*_*y*_ over the X and Y axis respectively;
- the deposition power *P*;
- the average intensity of the acquired image *I*_*mean*_;
- the powder flux coming out from the deposition nozzles;
- the *W-neighbors* and *W/2-neighbors* features.

These features are used to build a model for predicting the height of the deposited track *H*. As part of the pre-processing, all features have been rescaled to be in the range [0,1] using the min-max normalization:


(2) $$\begin{array}{l} X_{\text{scaled}}=\frac{X-\min\left(X\right)}{\max\left(X\right)-\min\left(X\right)}. \end{array}$$

The simplest shapes, i.e., single tracks, V-tracks and spiral, are used for training and optimizing the model. 75% of the tracks for each geometry type are used for training, whereas 25% of them are in the validation set. One spiral track is used for training, one in the validation set. In order to prove the generalization capabilities of the model to more complex shapes, only these simpler geometries are used for building the predictive model. The random tracks are not seen by the model during the training and are used as a test set. A second experiment was performed, with just one random track (i.e. 25% of the available tracks) added to the train dataset, to investigate how much the model performance improves when a small amount of data coming from more complex shapes is added to the training.

The data has the form of a time series, and therefore Recurrent Neural Networks (RNN) are a suitable choice to predict the height of the track and to model over-deposition. RNNs are typically used to model time series (the track sequences in the present study), and LSTM networks have been the best-known variant for many years. They have been chosen in the present study as they provide a solution to the vanishing gradient problem of traditional recurrent neural networks and as they can handle long-term dependencies (Hochreiter and Schmidhuber, 1997). Gated Recurrent Units (GRU) often achieve better results than LSTMs with small amounts of data due to their lower complexity, as they only have two gates instead of three (Chung et al., 2014). However, some preliminary tests showed no improvement in the model performance using GRUs, therefore a LSTM architecture has been used in the present study. The architecture was implemented using Keras (Chollet, 2015). A grid search was implemented to determine the best hyperparameters of the model. The following parameters have been tested: batch size (32, 64, 96), LSTM nodes (96, 128, 160), LSTM layers (1, 2), learning rate (0.0005, 0.001, 0.0015) and experiment (1 without random tracks, 2 with random tracks). All 108 combinations of hyperparameters have been tested, and the best 20 combinations have been analyzed. The best model performance has been reached with a batch size of 64. The number of LSTM nodes, LSTM layers and learning rate have a small effect on the model performance. The model used in the present paper comprises one LSTM layer of 128 nodes, which are trained for 30 epochs with a learning rate of 0.001, with a learning rate decay of 0.8. The values of the features for 20 deposition steps (*t*, *t*−1, … *t*−19) are given as input to the LSTM to predict the height at the time *t*. The Mean Squared Error (MSE) is used as a loss function during training and subsequently as a metric to estimate the performance of the model. The relative error (RE) on the prediction has also been used to compare the results on different geometries. This metric is defined as:


(3) $$\begin{array}{l} RE\%=\frac{\text{mean absolute error}}{\langle H\rangle }\cdot 100 \end{array}$$

The code developed for the present study is available at this link.

Permutation feature importance has been used to evaluate the effect of each feature on the results. This approach was firstly introduced with random forest (Breiman, 2001) and consists in the evaluation of the performance loss of the model when a single feature is shuffled randomly. A bigger performance loss indicates a bigger importance of the specific feature.

## 3. Results

**Figure 4:**
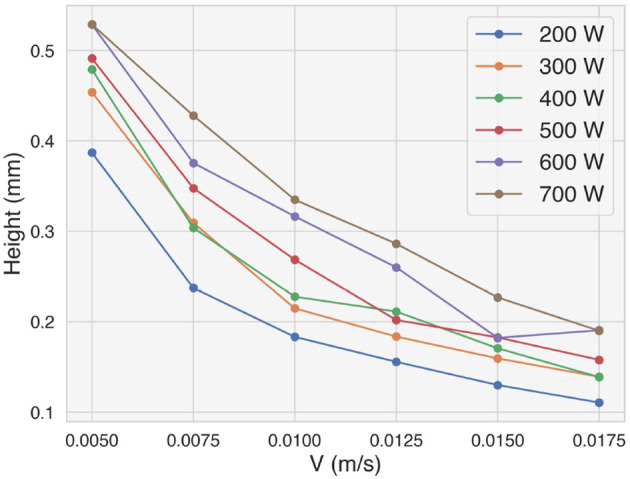
Average height of the deposited single tracks as a function of the nominal deposition speed for different deposition powers.

The deposition of single tracks has been done on a wide range of values for the deposition power and speed. Figure 4 shows the average height of each track as a function of the deposition speed. Different data series account for the different deposition power used. The average height has been evaluated only on the stable portions of the tracks, i.e. the beginning and the end of the tracks, where the deposition head accelerates and decelerates, have been excluded. The height decreases with the deposition speed and increases with the deposition power used. A lower deposition speed leads to a longer time spent on a specific portion of the substrate, resulting in more deposited material. A higher power leads to a higher energy density of the melt pool, which also leads to a higher track being deposited.

**Figure 5:**
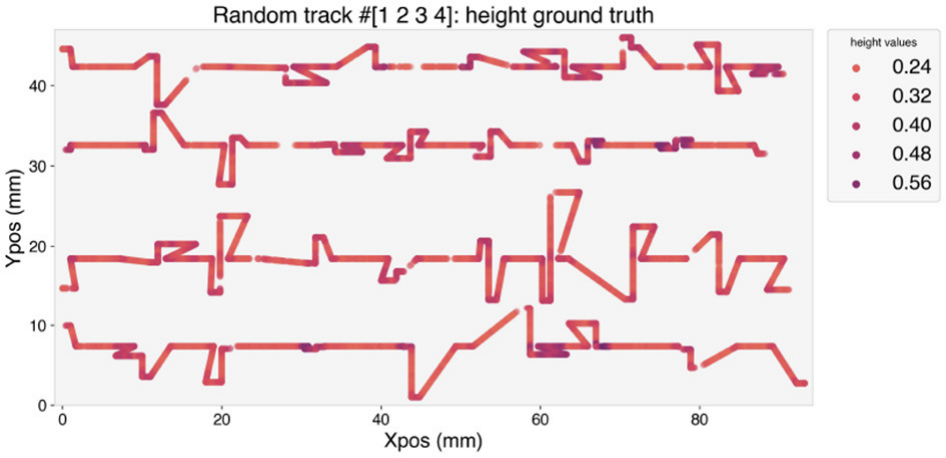
Height of the deposited random tracks.

The height along the trajectory of the random tracks is depicted in Figure 5. A darker color indicates higher values of the height. Over-deposition occurs when the deposition head changes its direction, and it is clearly visible from the higher values of *H* measured at the corners of the trajectory.

**Figure 6:**
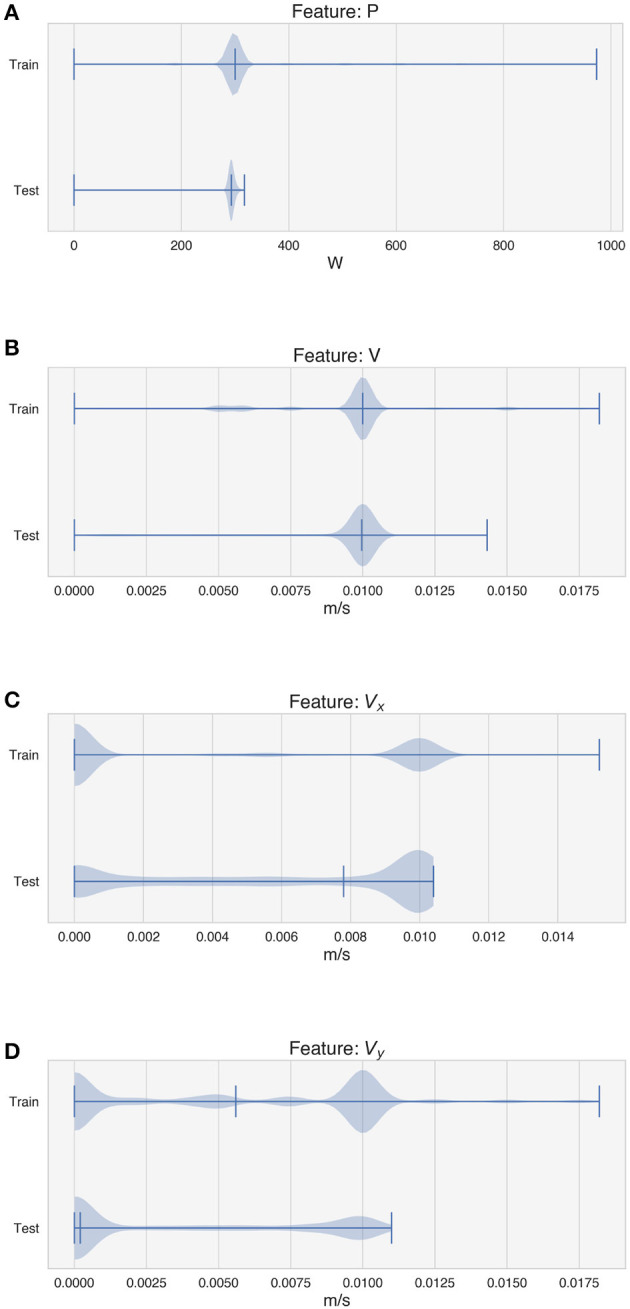
Distribution of the features used by the model for training and testing. **(A)** Deposition power. **(B)** Deposition speed. **(C)** Component of the speed vector along the x-axis. **(D)** Component of the speed vector along the y-axis.

**Figure 7:**
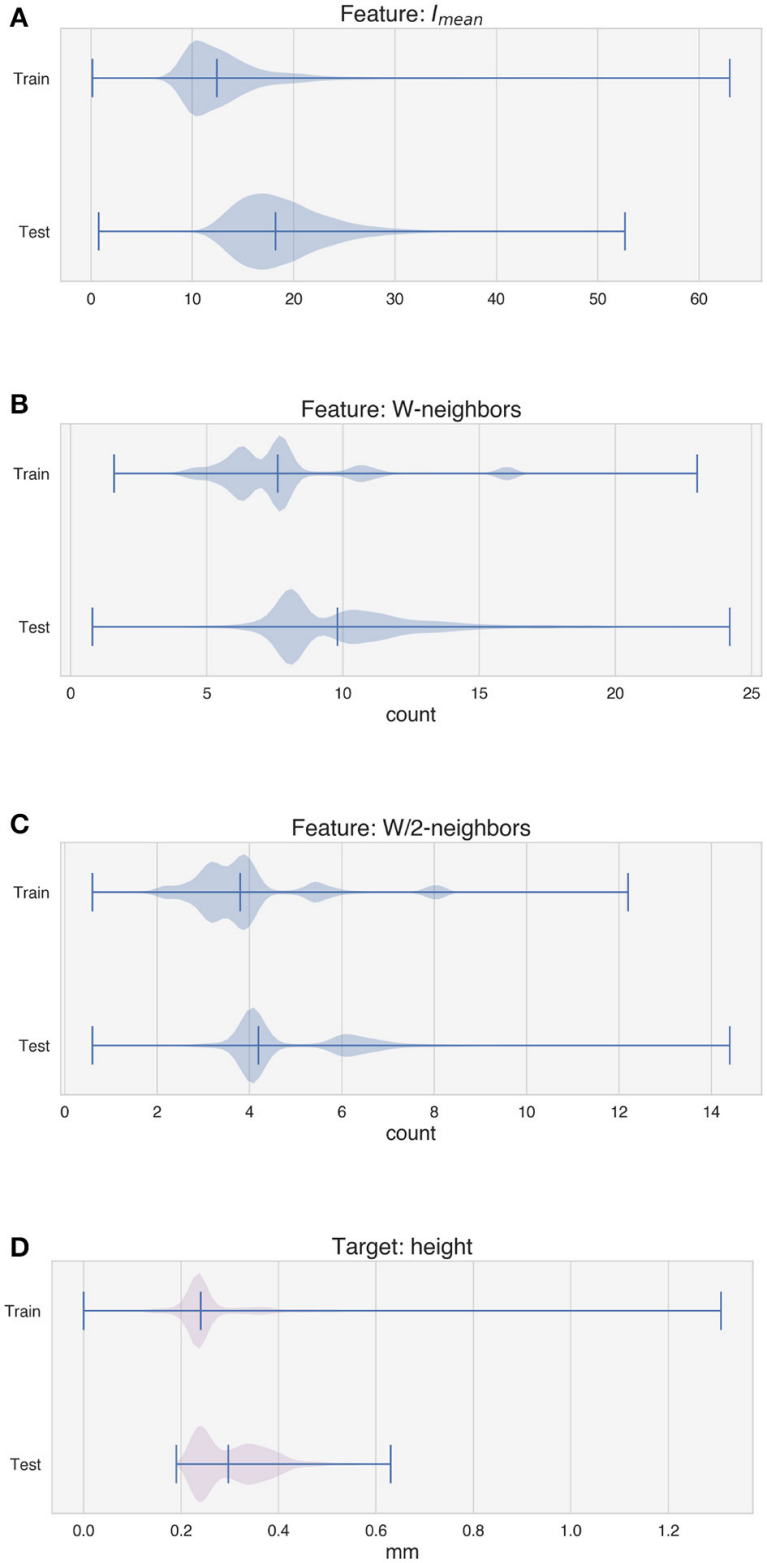
Distribution of the features used by the model for training and testing and distribution of the target. **(A)** Average intensity of the melt pool images. **(B)** W-neighbors. **(C)** W/2-neighbors. **(D)** Height of the tracks.

Figures 6, 7 depict the distribution of the features used for training and testing the model for experiment 1, i.e., with the random tracks used solely for testing purposes. The violin plots show that the distribution of the features between the two datasets is similar, although some differences can be observed in *V*_*x*_ and *V*_*y*_ (Figures 6C, D), in the average intensity *I*_*mean*_ (Figure 7A) and in the features W-neighbors and W/2-neighbors (Figures 7B, C). The distribution of the height of the tracks, the target of the LSTM model, also shows that higher values of *H* are more represented in the test dataset. The differences in the feature and target distributions arise from the properties of the trajectory of the random tracks compared to the simpler geometries. The angles at the corners in the train dataset can only take on certain specific values (20°, 45°, 90°), while many more values are present in the test dataset. As the random tracks contain more corners, the amount of data points showing higher values of *I*_*mean*_ as well as higher over-deposition (i.e., higher values of *H*) is also higher in the test dataset. Moreover, the randomness of the trajectory also accounts for differences in W-neighbors and W/2-neighbors, where a higher frequency of higher values is present in the test dataset.

**Figure 8:**
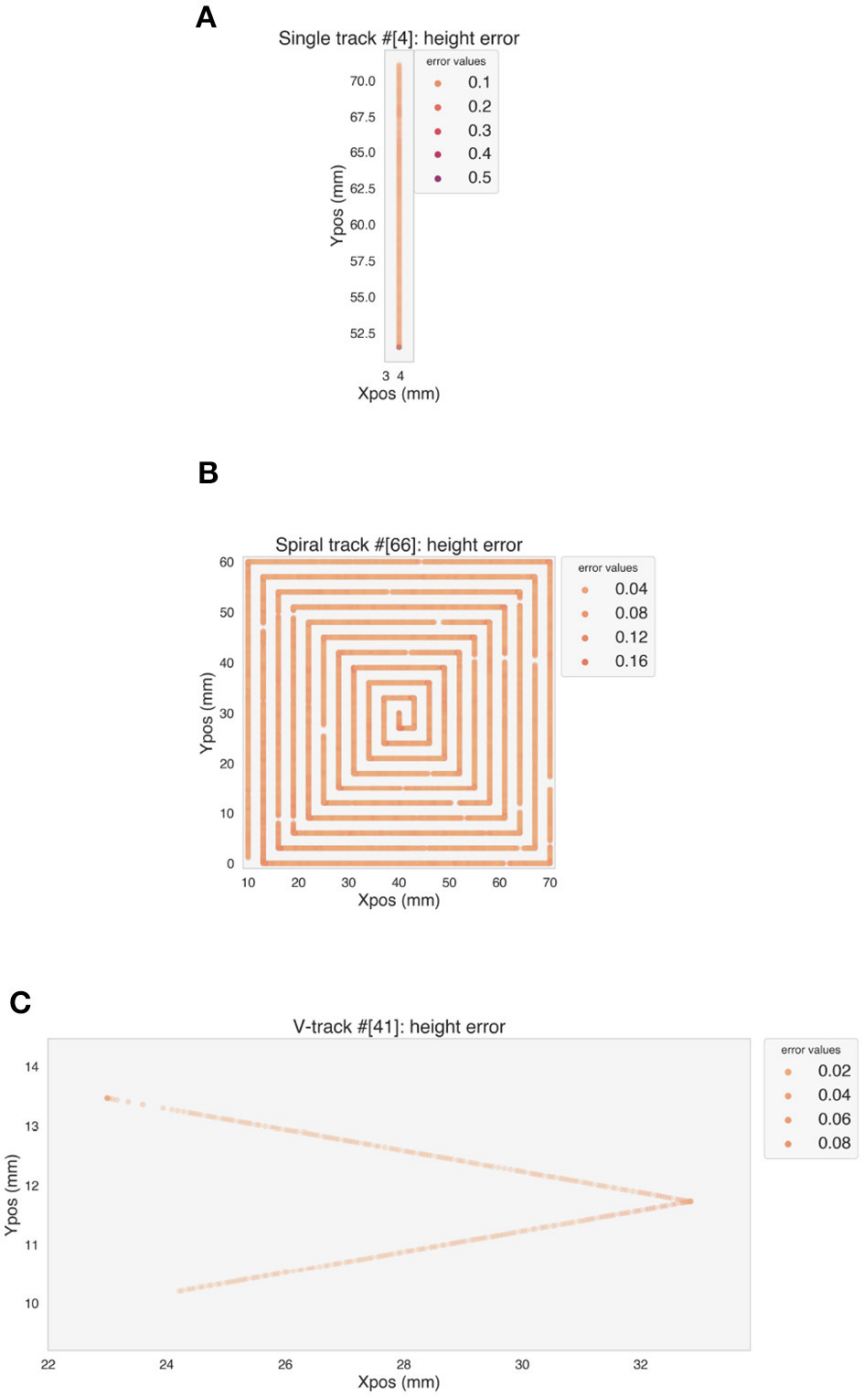
Error on the predicted height values on one example of validation track for each simple geometry. **(A)** Single track. **(B)** Spiral. **(C)** V-track.

**Figure 9:**
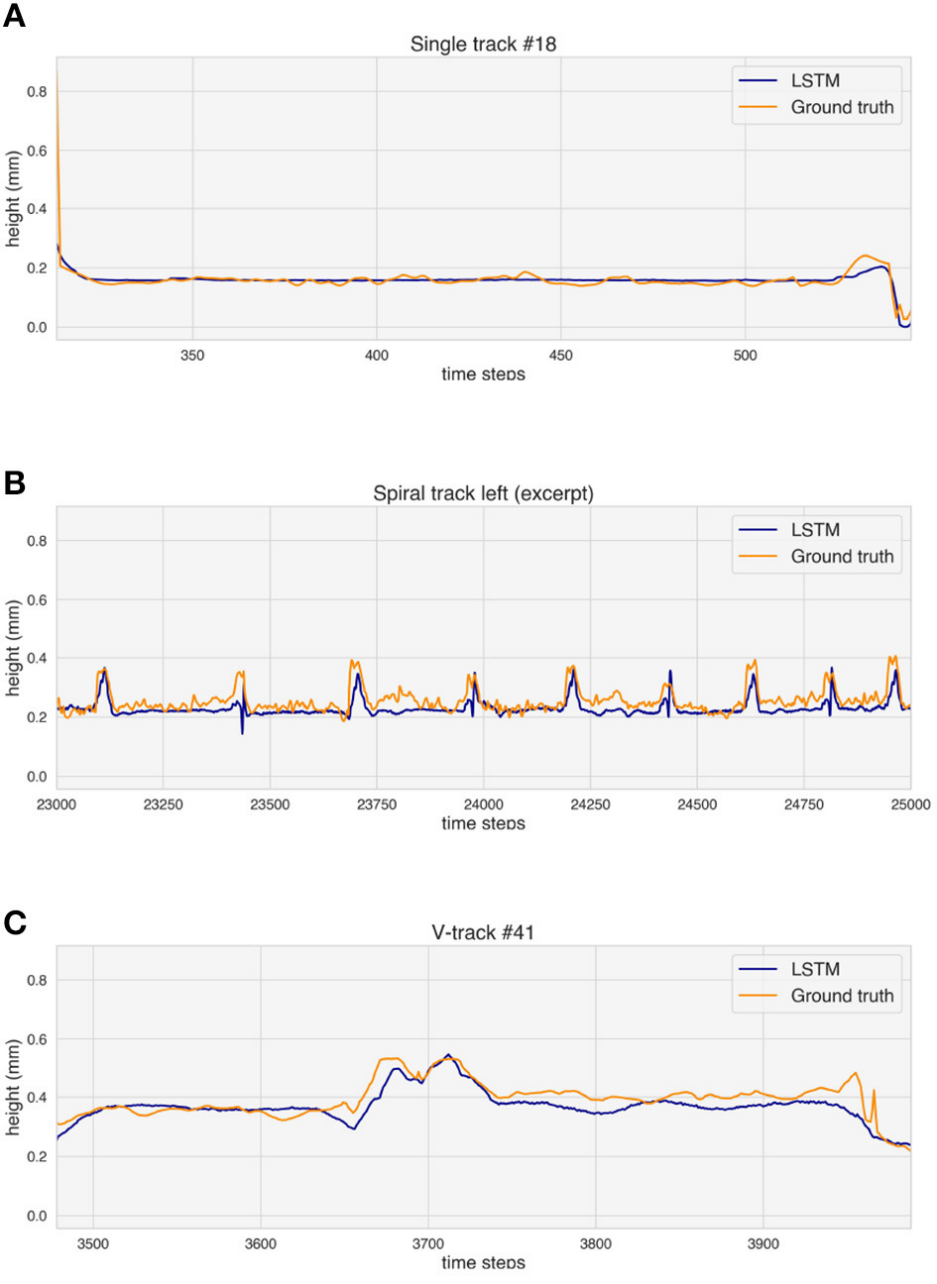
Ground truth and height prediction for one example of validation track for each simple geometry. **(A)** Single track. **(B)** Spiral (excerpt). **(C)** V-track.

Figure 8 shows the MSE for each predicted point of one validation track for each simple geometry. The LSTM model is able to predict the stable part of the tracks with a low error. An increase in the error appears at the corners, where over-deposition occurs, as well as at the beginning (Figure 8C) or at the end (Figure 9A) of a track, where the deposition speed is not stable, but in general the model can generalize well to test data, despite the different distributions of features and target.

Figure 9 analyzes the observed differences between ground truth and predicted height values for the selected validation tracks. The predicted values rise where over-deposition occurs, but a delay in the increase can be seen both for the spiral (Figure 9B) and the V-track (Figure 9C).

**Figure 10:**
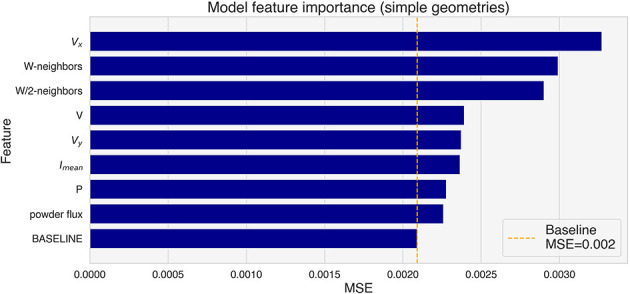
Feature importance for the tracks in the validation set (simple geometries).

The effect of each feature on the prediction has been estimated with permutation feature importance on the validation tracks, and the results are shown in Figure 10. *V*_*x*_ shows the higher importance, followed by both the neighbors features. It is important to note that correlated features can result in a reduced importance in the analysis (Kaneko, 2022). This means that the importance of some of the most important features, namely *V*_*x*_, *V*_*y*_, W-neighbors and W/2-neighbors is probably underestimated.

**Figure 11:**
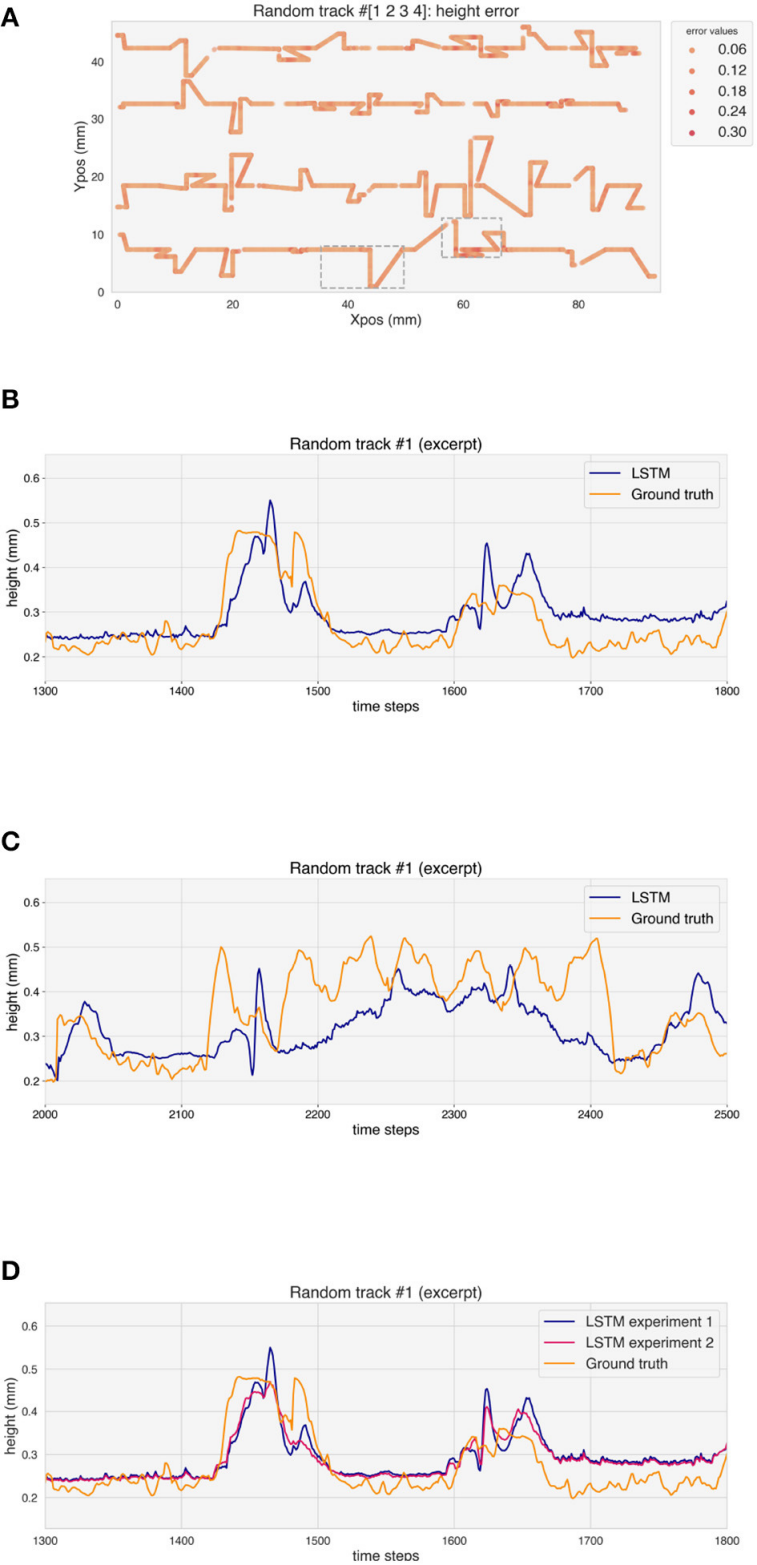
**(A)** Error on the height prediction for the test random tracks. **(B, C)** Ground truth and height prediction for one example of test random track (excerpt). **(D)** Ground truth and height prediction for both experiments for one example of test random track (excerpt).

The performance of the model has been evaluated on the test random tracks. Figure 11A shows the MSE along the random tracks. Also in this case, the stationary parts of the tracks show lower errors with respect to the corners, where over-deposition occurs. The predicted height of the tracks increases at corners, but also in this case some delay can be observed (Figure 11B). Moreover, the LSTM seems to overestimate the over-deposition. Where the deposition head passes multiple times in a small region, the LSTM fails to follow the height evolution of the track and underestimates *H* (Figure 11C).

**Figure 12:**
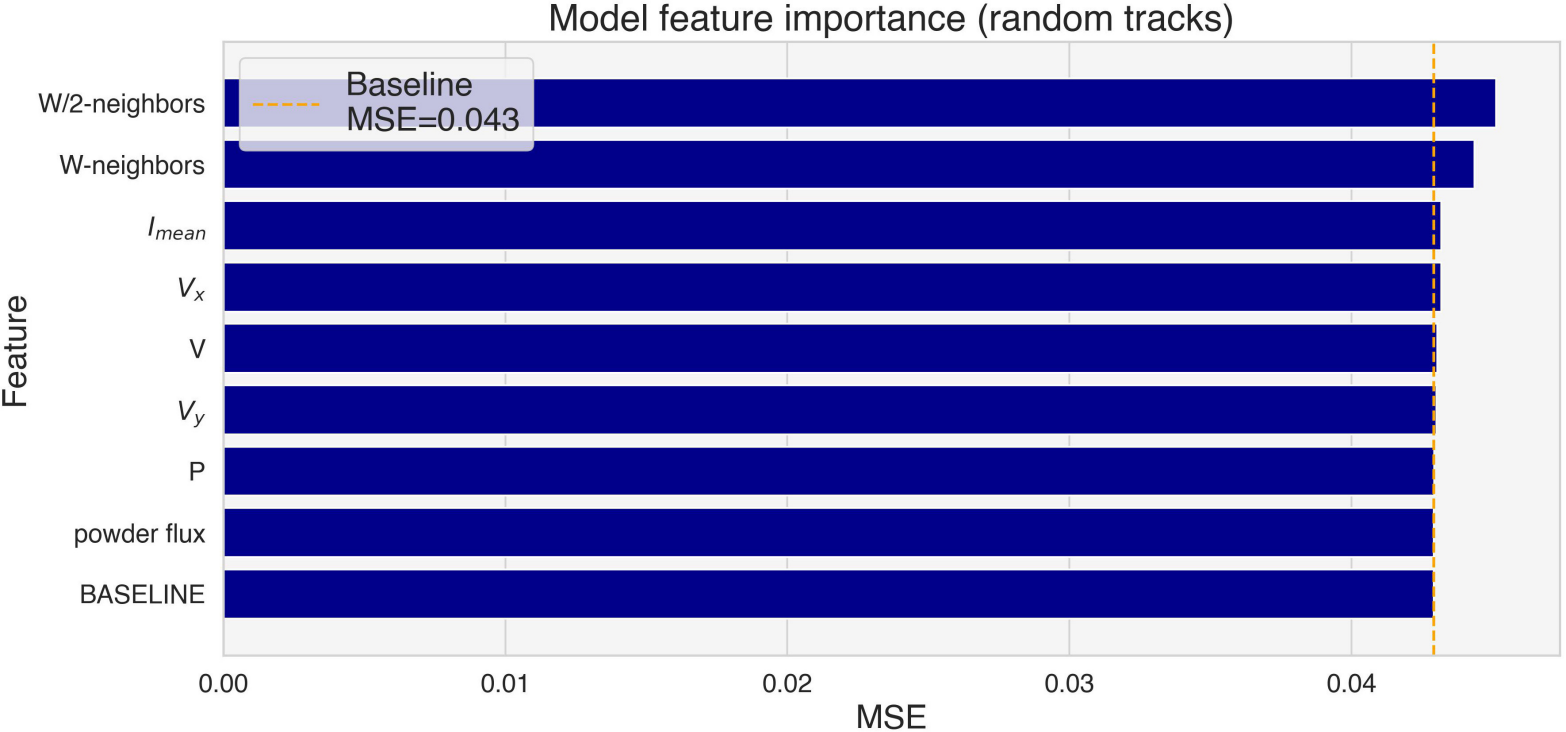
Feature importance for the test tracks (random tracks).

The feature importance analysis has been repeated on the test tracks, where the W-neighbors and W/2-neighbors features show the biggest importance (Figure 12). It is important to note that all random tracks have been deposited with the same powder flux and nominal power. The random shuffling of those features has therefore no effect, and they have been included only for completeness.

Table 2 reports the RE for the different geometries for the two experiments performed, i.e. without and with one random track in the training test, respectively. The relative error on the predicted height for the validation tracks is 7.06% for experiment 1. This value rises to 11.13% for the random tracks. When one random track is included in the training dataset, the error on the validation tracks rises to 7.83%, while the error on the random tracks decreases to 9.37%. Moreover, the overestimation of the over-deposition for the test tracks is reduced in experiment 2, as it can be seen in Figure 11D.

**Table 2:** Error on the validation and test tracks for experiments 1 and 2 (without and with one random track in the training data, respectively).

**Track type**	**Experiment 1**	**Experiment 2**
	**RE %**	**RE %**
Validation set	7.06%	7.83%
Single tracks	7.24%	7.82%
V-tracks	6.68%	6.82%
Spiral	7.12%	8.04%
Random tracks	**11.13%**	**9.37%**

The bold values indicate errors on the test datasets.

## 4. Discussion

The LSTM model is able to model over-deposition, as shown by the increase in the predicted height at the corner of the trajectories. There are some delays, which can be explained by the fact that the model only receives information about the last twenty deposition steps. The only two features that can account for the future development of the trajectory to some degree are W-neighbors and W/2-neighbors, which are designed to consider trajectory points not deposited yet. Therefore, the model only has limited information about the upcoming corner in the trajectory and the prediction suffers from a time lag. One way to overcome this issue is to give the model the values of certain features for future time steps (i.e. *t*+1, *t*+2 and so on). This would be possible for features such as the speed and the neighbors features, as the trajectory of the deposition head is known before the deposition starts.

The deposition speed and power determine the average height of the straight sections of the tracks, as shown in Figure 4. However, the speed and neighbors features show a bigger relevance to the model to predict over-deposition. This is consistent with the fact that the lower speed of the deposition head at the corners of the trajectory contributes significantly to the deposition of more material. Moreover, *V*_*x*_, *V*_*y*_ and the neighbors features consider to some extent the geometric properties of the trajectory around the section being analyzed.

The relative error of the LSTM rises from an average of 7.06% for the simple geometries to 11.13% when predicting the random tracks, which are never seen during the training (experiment 1). A rise in the error is to be expected, as the distribution of the features in the random tracks shows some differences with respect to the simple geometries. This phenomenon is known as covariate shift (Quinonero-Candela et al., 2008) and it is quite common in machine learning applications. The increase in the error when predicting the random tracks is small, and it shows that the model is robust and can generalize well to more complex geometries never seen during training. This is very promising for the application of the method to multi-layer objects, where a good generalization is critical, since additive manufacturing is often used for rapid prototyping and repair, meaning that the geometries of the manufactured objects are constantly changing.

The decrease of the RE from 11.13% to 9.37% for experiment 2, i.e. when one random track is added to the training set, shows that the addition of a small amount of data coming from more complicated geometries is sufficient to successfully limit the performance degradation of the model. In this case, the difference in the prediction capability of the model on the single geometries and the random tracks is reduced to less than 2%. This is also an encouraging result for extending the method to more complex geometries and multi-layer objects.

Similarly to the case of the single geometries, the prediction of the LSTM model suffers from a time-lag when predicting the height at the corners of the random tracks, which can also be traced back to the features given to the model as an input. Moreover, the model overestimates the over-deposition, which can be caused by the differences between the training and test set, such as the presence of many more values for the angles at the corners of the tracks. The reduction of this overestimation for experiment 2, where one random track is added to the training dataset, supports this hypothesis. In some cases, the model fails to follow the evolution of the height of the tracks in regions where the track sections come very close to one another, a situation which is not present in the training dataset.

The results suggest that the choice of the simple geometries used for building the training dataset is crucial for modeling the LMD deposition process. This will be even more important when more complex objects and multi-layer structures are considered. In this study, we focused on random tracks designed to investigate over-deposition, but further work is needed to generalize the approach.

The system presented in this study and the hardware used have not been optimized for making predictions in real-time during the deposition. Nonetheless, the model can make a prediction in ca. 50 ms on the CPU of an Apple M1 Max with 64 GB of RAM, which means that one prediction every 10 data acquisition steps is possible. Moreover, the system predicts only the height at one specific time *t*, but an extension of the approach for prediction of the height of multiple time steps in the future (i.e. *t*, *t*+1, and so on) is possible and could be beneficial in real-time settings.

## 5. Conclusions

In this study we investigated the generalization capabilities of LSTM networks for the task of predicting the geometry of a deposited piece in laser metal deposition. In fact, one of the strengths of LMD lies in its rapid prototyping capabilities, which means that the object to be deposited has a new geometry, which realistically will not be present in the dataset used for training a machine learning model. The proposed pipeline of data acquisition, feature engineering and model architecture can effectively model over-deposition for simple geometries like single tracks, V-tracks and spirals, but also generalizes well to more complex shapes such as random tracks never seen during the training, with only a small reduction in the performance. Moreover, adding a small amount of data from the random tracks further reduces the performance loss to less than 2% with respect to the simple geometries. The possibility to train the model on a set of basic geometries and then to generalize to different shapes could be incredibly useful for LMD practitioners. The results are very promising for the extension of the approach to multi-layer objects and for being used in a closed-loop control with command parameters and process feedbacks, to optimize the deposition geometry on-line.

## Data availability statement

Publicly available datasets were analyzed in this study. These data can be found here: Zenodo, https://zenodo.org/search?page=1&size=20&q=Baraldo&type=dataset.

## Author contributions

MP: design of the study, code development, and writing of the original draft. RJ: code development, visualization, and review of the original draft. SB: design of the study, experimental campaign, review and discussion of the results, and review of the original draft. AV and BP: review and discussion of the results, review of the original draft, and funding acquisition. All authors contributed to manuscript revision, read, and approved the submitted version.
